# How to Set Up a Molecular Pathology Lab: A Guide for Pathologists

**DOI:** 10.5146/tjpath.2020.01488

**Published:** 2020-09-15

**Authors:** Anıl Aysal, Burçin Pehlivanoğlu, Sümeyye Ekmekci, Betül Gündoğdu

**Affiliations:** Department of Molecular Pathology, Dokuz Eylul University, Graduate School of Health Sciences, Izmir, Turkey

**Keywords:** Molecular pathology laboratory, Laboratory setup, Guide

## Abstract

In today’s pathology practice, pathologists combine molecular tests with conventional histopathological methods. Pathology laboratories should therefore be designed and operated in accordance with the requirements of molecular testing procedures. While the specifics of the requirements may vary depending on the spectrum of the tests that will be performed, there are several basic criteria that need to be fulfilled for standardization. Adequate space, appropriate equipment and qualified personnel are required to establish a molecular pathology laboratory. One of the most important points that should be taken into consideration while designing a molecular pathology laboratory is to create a plan to prevent contamination. As molecular diagnosis has a major role in treatment decisions, the management of the molecular pathology laboratory is of utmost importance. In this review, the criteria required to establish an optimal molecular pathology laboratory will be reviewed.

## INTRODUCTION

The effects of diseases on the human body were first documented by ancient Egyptians however, the concept of organ-specific disease and anatomic pathology have begun to evolve only in the last few centuries, and the alterations at the tissue and cellular level have gained attention following the invention of the microscope (1). The molecular pathology era has begun with the integration of molecular tests into pathology practice, especially in diagnosis of solid tumors and hematological malignancies, as a part of the improvements in molecular sciences that had been led by the completion of the Human Genome Project in early 2000s. Pathologists now have a critical role of morphomolecular assessment in this new generation medicine era (2) and therefore have critical impact on the preanalytical phase of molecular testing (3). Given that pathologists combine molecular tests with conventional pathological evaluation methods, pathology laboratories should be designed and operated in accordance with the requirements of molecular testing procedures.

Adequate space, appropriate equipment and qualified personnel are required to establish a molecular pathology laboratory. While the specifics of the requirements may vary depending on the spectrum of the tests that will be performed, there are several basic criteria that need to be fulfilled for standardization. In this paper, the criteria required to establish a molecular pathology laboratory will be reviewed.

### Required Physical Conditions

One of the most important points that should be taken into consideration while designing a molecular pathology laboratory is to create a plan to prevent contamination. Polymerase chain reaction (PCR)-based methods are especially susceptible to contamination (4). To obtain a large number of copies from a very small amount of the target sequence with the PCR method provides an important diagnostic advantage, but this ability also leads to false results in case of contamination. False positive results may occur due to contamination from sample to sample, transport of amplicons from the previous amplification of the same target, cross-contamination of different reactions prepared simultaneously, and contamination of the reagents with DNA templates (5). In real-time PCR methods; when the PCR reactions are finished with fluorescence-based detection techniques, the analysis is also completed at the same time. Since the PCR products do not need to be reprocessed, the reaction tubes or closed plates are not opened and the amplicon transport does not occur (4). The laboratories using real-time PCR methods therefore have less risk of contamination (5).

Main procedures performed in a molecular pathology laboratory using PCR-based methods are pre-PCR procedures (sample preparation, PCR preparation) and post-PCR procedures (performing PCR and post-PCR analysis) (4). It is critical to perform these operations in areas separated as “clean” and “dirty”. The “clean” area represents the area where all pre-PCR procedures (such as microdissection, DNA/RNA extraction, PCR preparation) are performed, and the “dirty” area represents the area where all post-PCR products (amplicons) are processed. The staff and researchers should keep all reagents, materials and equipment used in these areas separate at all times and never move them back from the dirty area to the clean area (6). Contamination is significantly reduced by physical separation of the clean and dirty areas and by doing pre-PCR and post-PCR activities in separate rooms. Therefore, planning at least two separate rooms is essential while designing a molecular pathology laboratory. It is recommended to perform sample preparation steps such as nucleic acid isolation in the pre-PCR laboratory, and to perform PCR reactions and other post-PCR procedures in the post-PCR laboratory (4). However, if there is sufficient space, four separate rooms are recommended for the **preparation of reagents**, **sample preparation**, **PCR step** and **post-PCR steps** for an ideal molecular pathology laboratory. Each room must have its own equipment, protective clothing and consumables, and there should be no material/equipment transport between the rooms (7). The requirements for laboratory design may vary according to the method used. For example, as mentioned previously, 3 rooms may be ideal in a laboratory where a real-time PCR method is applied, since post-PCR analysis is not necessary in the real-time PCR method (5).

**Reagent preparation room** is the room where reagent stocks are prepared and then divided into a certain number of small usable parts (aliquoted), and the reaction mixes are prepared. This room should be free of any biological materials such as DNA/RNA extracts, PCR products, etc. **The sample separation room** is where the nucleic acid isolation is performed and the isolated samples are added to the PCR reaction mixes (5). This room is also called a “low copy” room, as the number of copies has not yet been amplified by PCR (8). Ideally, it is recommended to perform the steps of nucleic acid isolation and addition of isolated samples to the PCR reaction mixes in separate rooms, but these two steps are usually performed in the same room but in different areas/compartments since most laboratories do not have sufficient space (5). Preparation of the PCR reactions in a laminar flow biosafety cabinet ensures that the area remains clean (9). **The amplification (PCR) room **is where PCR devices are located and the amplification steps are performed, and the **post-PCR room **is where the analysis of PCR products by gel electrophoresis, sequencing, nested-PCR, etc., methods are carried out. These two rooms constitute contaminated-dirty rooms and no equipment or materials used in these rooms should be used in other rooms (5). These rooms are also called “high copy” rooms (8). In the PCR applications such as real-time PCR where single-stage PCR reactions are sufficient and tubes with PCR products are not required to be opened, PCR devices can be placed in the post-PCR room. However, in the laboratories using PCR applications such as nested PCR, etc., where multiple PCR reaction steps are required and the tubes must be opened, PCR devices should be placed in a separate room/area (9). In the amplification phase, the primary and secondary PCR steps (if any), should be separated according to the physical state of the laboratory, preferably in separate rooms. If this is not possible, they should be performed in separate compartments and on separate PCR devices (7). Next-generation sequencing applications also include one or more PCR amplification steps, which are similarly recommended to be performed in separate rooms/areas (5). Various recommendations about minimum room sizes can be found in international guidelines. For example, according to the field planning criteria of the United States of America Military Health System Pathology and Clinical Laboratories guide, the reagent preparation room should be at least 120 sq ft (approx. 33.5 m2) and the amplification room should be 240 sq ft (approx. 22.3 m2) in size (10). The relevant guide published by the Republic of Turkey Ministry of Health is detailed below.

### Workflow

**Figure 1:**
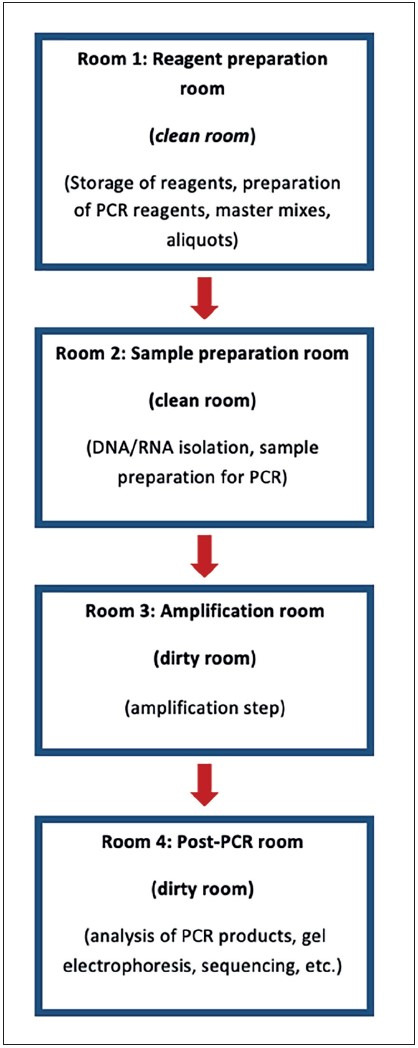
The Design and Workflow of an Ideal Molecular Pathology Laboratory *(Modified from references 4 and 8).*

The workflow in the molecular pathology laboratory must be unidirectional from the clean area to the dirty area (7). When laboratory personnel and researchers are required to move from dirty rooms to clean rooms, laboratory coats, gloves and all kinds of protective equipment should be changed and hands should be washed. No material should be carried from the dirty room to the clean room (5). To prevent the passage of personnel from the dirty room to the clean room, it is appropriate to have separate personnel working in each room or to perform pre-PCR and post-PCR procedures on different days (9). There are automated molecular pathology platform systems that provide automatic one-way workflow and isolate nucleic acid from the sample, combine the isolated DNA with amplification reagents, and perform the analysis, and their use is becoming increasingly common (5,11). The rooms and workflow that should be present in an ideal molecular pathology laboratory are shown in Figure 1. If all operations have to be performed in a single room, separate compartments/benches are required for the reagent preparation, sample preparation, PCR stage and post-PCR stages. The rule of unidirectional workflow from the clean compartments to the dirty compartments must be followed. If possible, sample preparation should be carried out in a laminar flow biosafety cabinet including UV light. In the absence of separate rooms, a timetable should be established in which the pre-PCR and post-PCR steps are performed at different times of the day (4).

### Ventilation

Circulating air between pre- and post-PCR laboratories is an important source of contamination in laboratories where techniques detecting very small amounts of DNA/RNA are used. Each laboratory should be ventilated separately and the air pressure must be adjusted separately. At positive pressure, the air pressure inside the room is higher than the air pressure outside the room, preventing the transport of unwanted substances from outside. Negative pressure, on the other hand, allows air to enter into the room and prevents the migration of the air to the surrounding rooms/laboratories. The doors must be kept closed to maintain the negative pressure. There should be slight positive pressure in the pre-PCR laboratory to prevent the entrance of contaminated air from outside, while the post-PCR laboratory should have a slight negative pressure to keep the air in and thus to prevent the escape of amplicons from the completed PCR samples. The ventilation of pre-PCR and post-PCR laboratories should be opened to different air channels and opened out from different locations (4).

### Ultra-Violet (UV) Irradiation

UV rays that cause DNA damage are useful for eliminating the contaminated DNA that may occur during addition of the DNA template. UV light can therefore be used to sterilize the pre-PCR laboratory. Since this method is based on cross-linking with thymidine residues, the base sequence of the target region plays a role in its success. In addition, the hydration status of the DNA has a significant effect on the UV resistance of the DNA. As dry-state DNA is more resistant to UV light, UV light is less effective in preventing contamination on dry laboratory surfaces (9). If UV light is going to be used on master mixes for decontamination, care must be taken to ensure that no dNTPs and enzymes are damaged in the UV light (8). The UV light source can be placed on the laboratory ceiling or bench and can be activated by a device on the exit door as the last person leaving the laboratory closes the outer door. If UV lights are used, UV-induced ozone must be removed by ventilation. Accumulation of deposits due to the precipitation of oxidation products on the glass of the bulb during radiation occurs and this reduces the effectiveness of the UV system. These deposits should be removed monthly and the performance of the UV bulbs must be strictly monitored (4).

The physical conditions required in molecular laboratories in Turkey are defined by the *“Guideline for Physical Infrastructural Standards of Medical Laboratories applying Molecular Tests”* published by the Republic of Turkey Ministry of Health (12). According to this guideline;

- Molecular diagnostic laboratories should have at least two, preferably three rooms, each with a minimum area of 15 square meters, physically separated from each other to allow unidirectional workflow (from preamplification to postamplification) and preferably with separate ventilation systems.
- These rooms are defined in parallel with those recommended in the literature and international guidelines, as a ‘pre-amplification laboratory’ where sample acceptance and nucleic acid extraction are performed, an ‘amplification laboratory’ where target amplification methods are applied, and a ‘post-amplification laboratory’ where analysis methods such as electrophoresis, DNA sequence analysis etc. take place after amplification.
- In order to prevent contamination, the issues of providing a clean and preferably separate airflow, preparation and storage of all reagents and chemicals in their own areas, use of separate devices and materials for each laboratory area (freezers, refrigerators, cabinets, centrifuges, water baths, vortex mixers, pipettes, pencils, timers, all kinds of consumables, etc.) are pointed out.
- It is emphasized that if only two rooms can be reserved for the molecular diagnosis laboratory, preamplification procedures and amplification / postamplification analyses should be performed in separate rooms.
- For each laboratory; the issues of the presence of single piece floor covering without pores, the presence of hand washing sinks, the provision of temperature and humidity monitoring, the use of UV irradiation systems (on counter tops and / or room ceilings) during non-working hours, the presence of sufficient storage space, the presence of a sufficient number of grounded electrical outlets and uninterruptible power supplies (UPS, generator, etc.) for the laboratory devices, and the placement of laboratory equipment to allow unidirectional workflow are pointed out.

### What to Do to Avoid Intermixing and Contamination of Samples

Molecular laboratory tests are generally quite sensitive and specific, providing very precise results (6). Even so, false positive or false negative results may sometimes occur. Control mechanisms that include the verification of the primer and probe sequences, checking and confirming whether the test conditions are optimal, and the use of negative controls (9) should be employed to reduce false results.

While amplification is generally a part of the molecular diagnostic method, current nucleic acid amplification methods are very sensitive with the capability of detecting even a single molecule. Although this seems to be an advantage, it should be kept in mind that the contaminated DNA molecule may also be amplified, causing false positive results. Therefore, prevention of contamination must be a priority in molecular pathology laboratories (9).

Cross-contamination is one of the sources of error and contamination in the pathology laboratory and may occur at any stage of the tissue processing process, such as during macroscopic and/or microscopic evaluation, or during DNA extraction, and may cause false positive or negative results. Microorganisms (viruses, bacteria, etc.) may also be transferred from one case to another during these processes. Immunohistochemical staining with ABH blood group antibodies, microdissection, and microsatellite instability analysis can be performed to prevent and detect cross-contamination (2,13). As processing of the samples from different patients occurs in the same area with recurrent use of several instruments (e.g., microtome blade, water bath) in pathology laboratories, precautions such as using a new blade for each sample, washing the blade with DNA decontamination solution, and/or sectioning of an empty paraffin block between samples (called the “*sandwich model”*) are used by various laboratories to reduce the risk of cross-contamination (13). However, cross-contamination rates have been reported to be around 3% (0-8.8 %) despite these precautions (14).

The amplified DNA from the positive reactions in the previous test, when the reaction tubes are opened after amplification, is the source of contamination for the subsequent tests (9). Also, amplification reactions are exposed to contamination from other patients’ samples and the target-containing plasmid (15). Samples may contaminate the laboratory environment while pipetting and the risk of contamination increases if multiple samples are run together. Positive controls studied in the test are risk sources for contamination as well. Clothing, laboratory waste and/or uncleaned tables may contain contaminating nucleic acids (9).

In order to prevent and control contamination, the appropriate physical conditions, architectural structure and design, meticulous application of laboratory techniques and environmental control protocols, and the workflow plan are essential. Using separate areas or rooms for pre-amplification, amplification and post-amplification stages with separate ventilation systems is an efficient way to prevent contamination, as discussed in detail in the “Physical Conditions” section above. The risk of contamination is lower in closed system devices. Only personnel in charge should be present in the test area. Every area/room should have its own equipment including laboratory coats and pipettes (4,6,15–17).

Minimum aerosolization while opening tubes is also necessary to prevent transport between samples (15).

Cleaning before and after each procedure should be carried out by using nucleic acid removers. For example, washing with 10% bleach that is freshly prepared and rinsing with 70% ethanol can be performed (16). Thus, both biologically hazardous substances and nucleic acids that may be sources of contamination can be removed (15). Adding enzymes such as uracil DNA glycosylase, which break down DNA, into the amplified DNA to exchange some or all of the thymidine with uracil in the reaction products can prevent contamination biochemically (18). In addition, contamination can be prevented or reduced by discarding unopened tubes in the last stage, using pipettes with positive pressure displacements, not talking during tests such as PCR, and regular exposure of laboratory devices to UV radiation (4,6). Aliquoting the reagents for each run is another precaution for the prevention of contamination. Patient samples and positive controls should be the last to be put into reaction tubes to reduce the risk of transport of the nucleic acids. If positive controls are going to be used, the lowest dilutions should be preferred (15).

Water or DNA-free buffers can be used as negative controls to detect and monitor contamination. The control tube should contain all materials for all stages of the test, like any other sample. A positive result detected in the negative control indicates the possibility of contamination (4,9). The adequacy control of chemical sterilization (such as uracil glycosylase protocols) can be done by incorporating a small amount of amplicon into a negative control (15). Surface and equipment contamination can be checked by swab samples from the laboratory surfaces where the test is carried out with damp filter papers, and a positive result in the swab sample indicates the presence of contamination. In addition, more than expected positivity rates of a given test may indicate contamination (4,9).

As RNA is more reactive than DNA and is vulnerable to RNAses that are present in all cells, prevention of RNAse contamination is very important in RNA-based molecular tests. RNases are resistant to metal chelating agents and can persist even after prolonged boiling or autoclaving. The most common sources of exogenous RNAse contamination are contaminated buffers and automatic pipettors. Also, all laboratory surfaces and glassware can be contaminated with RNAse from the laboratory personnel’s skin, hair, etc. Wearing gloves and changing them frequently during all stages of the test, use of separate laboratory equipment for RNA based tests, aliquoting small amounts of buffers, use of RNAse inhibitors (DEPC, etc.), and use of RNAase-free solutions and tubes are among the laboratory precautions to prevent RNAase contamination (19).

Preparation of a documented action plan in case of contamination is recommended. Most laboratories quarantine and/or destroy all contaminated reagents and consumables (15).

### Equipment

**Figure 2:**
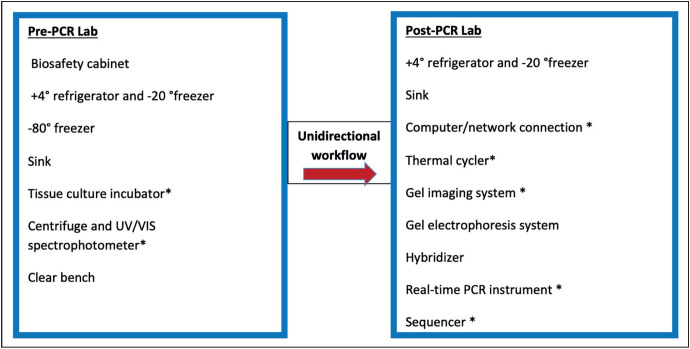
Equipment required in a two-room PCR laboratory (Modified from reference no. 4); *: must be kept on different benches/ in different areas.

Equipment and appliances may slightly differ between molecular pathology laboratories based on their testing profile. On the other hand, often a detailed inventory list (Table 1) is required to set up a molecular pathology laboratory and provide standard testing. It should also be noted here that equipment and consumables used in the routine pathology setting must be available in a molecular pathology laboratory, as well. It is also important to budget for service contracts for maintenance and repair (6). Unidirectional workflow should be taken into consideration while organizing/placing the equipment (Figure 2). The list of the appliances and equipment required in a molecular pathology laboratory provided by Republic of Turkey Ministry of Health is also a useful guide (Table 2) (12).

**Table 1:** The list of equipment and appliances required in a molecular pathology laboratory.

**Equipment**	**Purpose of Use**
Refrigerator	Storage of chemical kits and PCR products
-20°C and -80 °C freezer	Storage of tissue culture and frozen samples
Vortex mixer	Mixing small vials
Refrigerated and non-refrigerated centrifuge	Separation of components based on density
Thermal cycler	Polymerase chain reaction
Spectrophotometer	Nucleic acid quantification by measuring the amount of light absorbed by a sample
Sequencer	Sequencing
Microscope equipped with a camera system	Evaluation and archiving of test results (e.g., in situ hybridization)
Hybridizer	In situ hybridization
Nucleic acid quantification instrument	Nucleic acid quantification
Electrophoresis system	Analysis of PCR products
Ultraviolet light illuminator	To prevent contamination via ultraviolet light illumination
Autoclave	Sterilization
Incubator	Maintaining optimal conditions (e.g., temperature)
Uninterruptible power source	Emergency power source to maintain power input when/in case main power source fails
Liquid nitrogen tank	Snap freezing
Microwave	Heating, drying, vaporizing
pH meter	pH measurement
Microtome	Sectioning
Water bath	Incubating samples at a constant temperature
Slide oven	Slide drying, sterilization, maintaining constant/certain temperature levels
Tissue processor	Tissue processing
Tissue embedding machine	Tissue embedding
Computer, private network and software	Bioinformatics
Slide stainer	Staining
Coverslipper	To cover slides
Immunohistochemistry staining system	Immunohistochemical staining
Automated pipetting system	Transfer and handling of precise volumes of samples and reagents
Biosafety cabinet	To prevent contamination

**Table 2:** The list of appliances and equipment required in a molecular laboratory as set by the Republic of Turkey Ministry of Health (12).

**Required appliances and equipment**
-20 °C freezer +4 °C refrigerator Automated pipetting system (minimum two sets: 0,1-10 μl; 10-20 μl; 10-100 μl, 20-200 μl; 200-1000 μl) Class II biosafety cabinet PCR cabinet Dry heat block and/or water bath Spectrophotometer Thermal cycler Electrophoresis system Gel imaging system (ultraviolet light based or computed) Vortex mixer Refrigerated centrifuge (min. 5000 rpm) Microcentrifuge (min. 15000 rpm for 1.5 ml tubes) Magnetic stirrer Analytical balance pH meter Microwave Relevant plastic and glass instruments
**Recommended appliances and equipment**
-80 °C freezer Ice machine DNA sequencer Water deionizer system Hybridizer Fume hood

Instructions for calibration and maintenance should be kept in the laboratory as written guidelines. Calibration guidelines must include the schedule for calibration (e.g., daily, monthly etc.), instructions describing the steps of the calibration procedure, calibration material specifications, preparation and storage conditions, troubleshooting and documentation methods, maintenance guidelines, the schedule for maintenance, instructions for performing maintenance, and troubleshooting guidelines (20).

### Quality Assessment

Quality management is essential in all steps of pathology evaluation (i.e., pre-analytical, analytical and post-analytical) and a very important component in molecular pathology practice. However, as the details of quality management are beyond the scope of this review, only the basic principles will be mentioned here.

There are several regulatory guidelines, including standard operating procedure manuals, to be followed to set-up and/or manage a molecular pathology laboratory (12,17,20). The presence of errors affecting the accuracy of the results in a molecular pathology laboratory should be checked regularly under the supervision of the pathologist (“quality control; QC”) to prevent or minimize erroneous reports and provide the confidence that quality requirements will be fulfilled (“quality assurance; QA) (21).

Turn-around time and test result statistics should be checked and validated by using standard validation studies (22). For internal quality assessment (IQA), the use of control materials is recommended (see previous sections for details).

In addition to the internal precautions mentioned in the previous sections, external quality assessment (EQA) must also be performed at given intervals for specific types of tests, as it is the most critical stage of quality management. EQA, a measure of laboratory performance (23), has been shown to be helpful to improve molecular pathology laboratories (24), and EQA programs are the key elements of a laboratory’s QA framework (25). Regular participation in EQA is needed to verify and improve the quality of testing, as molecular pathology EQA schemes score the report and the test result (26,27). As a part of an EQA scheme, participants receive test samples and their results are then reviewed to check for errors.

The reports are scored and the participant laboratories gain the opportunity to improve their service. Laboratories across Europe are also required to have accreditation (26). Accreditation is a process in which an authorized independent body officially recognizes that the laboratory is competent to perform certain tasks, and may be considered the most effective system for QA as compliance with ISO standards is checked by accreditation bodies. Both accreditation and participation in EQA are recognized as effective and important tools to improve the accuracy and reliability of molecular testing (28).

## CONCLUSION

In conclusion, if the results obtained by molecular diagnostic tests are inaccurate due to any of the factors mentioned here, serious problems may arise which adversely affect the diagnosis and treatment decision. As molecular diagnosis has a major role in treatment decisions, especially for cancer patients, the management of the molecular pathology laboratory is of utmost importance.

## CONFLICT of INTEREST

The authors declare no conflict of interest.

## FUNDING

No funding to declare.
